# The Old and the New: Prospects for Non-Integrating Lentiviral Vector Technology

**DOI:** 10.3390/v12101103

**Published:** 2020-09-29

**Authors:** Luis Apolonia

**Affiliations:** Department of Infectious Diseases, School of Immunology and Microbial Sciences, King’s College London, London SE1 9RT, UK; luis.apolonia@kcl.ac.uk

**Keywords:** lentiviral vectors, non-integrating lentiviral vectors (NILVs), insertional mutagenesis, transgene expression, immunotherapy, CRISPR/Cas9

## Abstract

Lentiviral vectors have been developed and used in multiple gene and cell therapy applications. One of their main advantages over other vectors is the ability to integrate the genetic material into the genome of the host. However, this can also be a disadvantage as it may lead to insertional mutagenesis. To address this, non-integrating lentiviral vectors (NILVs) were developed. To generate NILVs, it is possible to introduce mutations in the viral enzyme integrase and/or mutations on the viral DNA recognised by integrase (the attachment sites). NILVs are able to stably express transgenes from episomal DNA in non-dividing cells or transiently if the target cells divide. It has been shown that these vectors are able to transduce multiple cell types and tissues. These characteristics make NILVs ideal vectors to use in vaccination and immunotherapies, among other applications. They also open future prospects for NILVs as tools for the delivery of CRISPR/Cas9 components, a recent revolutionary technology now widely used for gene editing and repair.

## 1. Lentiviral Vectors

Lentiviral vectors have been derived from human immunodeficiency virus-1 (HIV-1) and developed by segregating the necessary genetic information to form an infectious particle in different plasmids, while removing the capability for replication [1]. Earlier vector generations used three plasmids for vector production: the packaging plasmid that codes for all necessary viral proteins for the particle formation and delivery of the transgene into target cells, the envelope plasmid coding for a glycoprotein that allows for the recognition of target cells, and the transfer vector plasmid that codes for the transgene(s) of interest [2]. In the last generation of these vectors, the regulatory protein Rev was removed from the packaging plasmid and coded from a fourth, distinct plasmid [3].

The envelope plasmid codes for a glycoprotein from a different virus than HIV-1. A commonly used one is the heterologous envelope of vesicular stomatitis virus. This envelope protein allows for the recognition of many target cells and also stabilises the vector particle [4], although other viral proteins have been used. This process is called pseudotyping, and it is a topic covered in another review within this Special Issue [5].

The transfer vector is the viral particle genome coding for the transgene of interest. It is the only RNA in the cell that contains the HIV-1 packaging signal, enabling the specific packaging of this RNA into forming particles. This RNA contains the Rev-responsive element (RRE) that is recognised by the HIV-1 protein Rev, allowing for the nuclear export of unspliced RNA [6]. It also contains all necessary elements for reverse transcription and integration upon target cell transduction. In further developments of the transfer vector, the HIV-1 promoter that sits in the U3 region of the long terminal repeat (LTR) was removed, thus creating self-inactivating (SIN) vectors. These then require a heterologous promoter in the 5′LTR in place of the U3 region to allow the transcription of full-length transfer vector mRNA for incorporation into vector particles and do not rely on the viral protein Tat for transcription in the producer cells [7]. Additional modifications to the original transfer vector included the incorporation of a central polypurine tract (cPPT) that can increase the nuclear import of the viral DNA [8,9] and the incorporation of the post-translational regulatory element of the woodchuck hepatitis B virus (WPRE), shown to improve transgene expression levels by facilitating the nuclear export of transcripts that contain this element [10].

The packaging plasmid codes for structural and enzymatic proteins necessary for particle formation and further viral life steps until the transfer vector is integrated into the target cell genome. Gag (structural) polyproteins assemble into particles and recruit the vector genome. The enzymatic polyprotein Pol is composed of the viral protease that cleaves the polyproteins allowing for particle maturation, reverse transcriptase (RT), that reverse transcribes the RNA genome into double stranded DNA. This genetic material can then be integrated into the target cell genome by the viral integrase (IN) protein.

Once integrated, the viral genome becomes part of the target cell genome. It is replicated along all genes into the daughter cells during division, and it is transcribed like a normal cellular gene via its own promoter and polyA signal. Although the resulting RNA, unlike most cellular RNAs, does not contain introns, it can exit the nucleus into the cytoplasm, where it will be translated by the host cell machinery.

## 2. Insertional Mutagenesis Safety Concerns and the Rise of Non-Integrating LVs (NILVs)

An advantage of retroviral-based vectors compared with other viral-based vectors is the stable integration of the transgene into the host genome, thereby achieving sustained transgene expression even if the cells divide [1]. Nevertheless, this hallmark can also become problematic. In fact, there were several adverse events detected in clinical trials, later described, due to insertional mutagenesis [11,12]. In these trials, patients suffering from severe combined immunodeficiency (SCID)-X1 were successfully treated. This disease is an X-linked inherited disorder characterised by an early block to T and natural killer lymphocyte differentiation caused by mutations of the gene encoding the common cytokine receptor γ-chain subunit, important for the delivery of growth, survival, and differentiation signals to early lymphoid progenitors. The patients were then treated by using γ-retroviral vectors to deliver a corrected version of the gene to hematopoietic stem cells ex vivo, thus correcting the gene defect, with subsequent injection of the cells into the patients [11,12]. However, this procedure led to the origin of leukaemia in the treated patients. These events were characterised by insertional transactivation of oncogenes (such as LMO2) by the retroviral vector promoter that led to the uncontrolled clonal expansion of T cells in the patients [13,14,15]. Additional insertional mutagenesis events were observed in other gene therapy clinical trials using integrating retroviral vectors, such as in Wiskott–Aldrich syndrome (WAS), X-linked chronic granulomatous disease (CGD) or β-Thalassemia trials with loss of polyclonality of corrected cells. In the WAS trial, a γ-retroviral vector was used to deliver the corrected WAS protein gene to autologous haematopoietic stem cells, which were then transplanted into patients. This successful trial observed a sustained and partial or full resolution of the disease in 9 out of 10 patients. However, 7 patients developed acute leukaemias associated with dominant clones that harboured vector integrations within or close to the LMO2, MDS1 and MN1 genes’ locus [16]. CGD is characterised by a defect in the oxidative antimicrobial activity of phagocytes, resulting from mutations in the gp91(phox) gene. It was observed in this gene therapy clinical trial that the insertional activation of MDS1-EVI1, PRDM16 and SETBP1 increased the expansion of the gamma-retroviral vector-corrected cells in the treated individuals, potentiating the clinical benefit to patients [17]. However, later findings linked insertional activation of EVI1 to genomic instability, monosomy 7 and clonal progression toward myelodysplasia [18]. Similarly, a dominant, myeloid-based cell clone with the HMGA2 gene activated by insertion of the integrated lentiviral vector may have increased the therapeutic efficacy of the treatment in a β-Thalassemia gene therapy trial [19].

It was then evident that further vector development was necessary to reduce these adverse outcomes and the self-inactivating (SIN) vector design was shown to improve the biosafety profiles of these vectors [20,21]. Nonetheless, integration may not need to occur in certain applications, and a design where these vectors would not be able to integrate their genome into the target cells could increase the safety profile of lentiviral vectors even further.

## 3. Non-Integrating Lentiviral Vector Development

**Figure 1:**
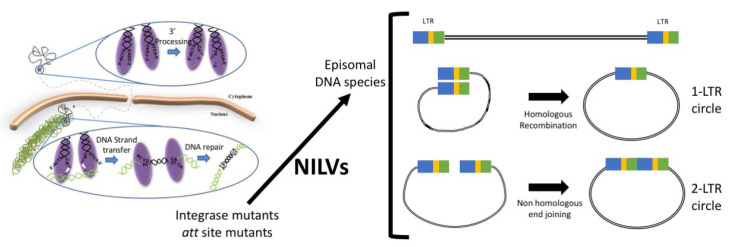
Schematic diagram of integration process, with 3′ processing depicted where the viral enzyme integrase removed a dinucleotide from the ends of the viral DNAs, while binding to the attachment (att) sites and the nucleophilic attack of the recessive ends to the target genome in a catalytic process called DNA strand transfer. Integrase and/or att mutations render these vectors non-integrative, giving origin to episomal vector DNA species that can either be linear or circular, with the latter being the origin of homologous recombination (originating one-LTR (long terminal repeat) circles) or products of non-homologous end-joining (circles with two LTRs).

Integration of the provirus into the host cell DNA is an important step of retroviruses. It ensures that the viral DNA is replicated and transmitted to the daughter cells during cell division. Four main components are necessary for integration to occur: the viral DNA, the target DNA, the viral enzyme integrase and host factors. The integrase viral enzyme is a proteolytic product of Pol and is composed of three domains: the N-terminal domain, which contains a zinc-binding motif [22] and binding of a zinc ion promotes multimerization of this protein [23]; the central domain that contains the catalytic core DD35E motif [24]; the C-terminal domain that has DNA-binding properties [25]. The host-factor LEDGF/p75 is utilised by integrase to facilitate host-cell chromatin binding and targeting lentiviral DNA integration [26,27]. Integration occurs in two catalytic steps: the viral integrase binds to the viral double-stranded DNA in the attachment (att) sites, which are two sets of conserved CA dinucleotides at the end of each of the viral LTRs, and removes the adjacent dinucleotide, in a process called 3′ processing [28]. In the second catalytic step, named strand transfer, integrase mediates a concerted nucleophilic attack by the 3′-hydroxyl residues of the viral DNA on phosphodiester bridges located on either side of the major groove in the target DNA and a transesterification reaction where the viral 3′ ends are ligated to the 5′-O-phosphate residues of the target DNA [29]. The final step is the removal of the unpaired 5′ ends of the viral DNA and the filling of the single-stranded gaps between the 5′ end of the viral DNA and the host DNA, done by the host DNA repair machinery. A schematic representation of these processes is depicted in Figure 1.

Interestingly, in addition to the integrated provirus, other extrachromosomal viral DNA species were identified early in retrovirus research [30,31]. These viral DNA molecules can either be linear or circular, with the latter being a product of homologous recombination between the viral LTRs (resulting in a DNA circle with only one LTR) or the result of non-homologous end-joining (hence, DNA circles with two LTRs) [32]. Additionally, there are other viral DNA circles produced by intramolecular integration [33]. The viral 2LTR circle molecules can only be formed in the nucleus and have been used as surrogates to assess nuclear entry. It was originally thought that these extrachromosomal molecules were by-products of integration and/or viral DNA dead ends. However, it was later proposed that these DNA molecules could support transcription [34,35,36]. Taking these observations together, Yanez-Munoz and colleagues tested lentiviral vectors that did not integrate their genome into host chromosomes and clearly demonstrated that these vectors maintained full capacity for transgene expression in target cells both in vitro and in vivo [37].

There are two main approaches to generate non-integrating lentiviral vectors: mutation of the viral enzyme responsible for the catalysis of this reaction or inhibiting the recognition of the viral DNA by this enzyme, by mutating or deleting the att sites. These mutations impede the integration of the viral DNA, thus increasing the episomal vector molecules (Figure 1).

With the discovery of the viral enzyme responsible for the stable integration of viral genome into the host chromosomes, and the molecular detail necessary for this reaction, including the catalytic site of this enzyme, the effect of mutations on the integration of vector DNA was investigated. Mutation of any amino acid of the catalytic triad (in HIV-1, these are D64, D116 and E152 residues) results in a catalytic-dead enzyme, therefore increasing the amount of unintegrated vector DNA while reducing the amount of DNA molecules that stably integrate into the host chromosomes up to 1000 fold [38]. Other individual IN mutations have a similar effect, although to a lesser extent (5- to 20-fold lower integration): H12A, N120L, Q148A, Q168L, F185A, W235E, K264R, K266R, and K273R [39,40,41]. 

Mutation or deletion of the att site also impairs IN-mediated integration of the viral DNA. These mutations can reduce integration up to 10000-fold [42]. However, the combination of mutations in the viral DNA and in the viral integrase can reduce illegitimate or background integration but does not act synergistically to lower integration much further [40,43].

Kantor and colleagues have recently reported another method to reduce integration of lentiviral vector DNA molecules. They postulated that reducing the formation of linear episomes would further decrease integrase-independent integration events. They show that deletion of the PPT (the viral sequence that acts as primer for plus-strand synthesis) from the vector genome resulted in the increase of 1LTR circle formation prior to the completion of RT, thus reducing the formation of linear DNA molecules and, concomitantly, the formation of 2LTR circles. This PPT-deleted vector reduced integration 10-fold and contributed additionally to a mutation in the viral IN to decrease integration a further ~3-fold [44]. The same laboratory has also reported the development of a stable NILV packaging cell line that may aid in the production of clinical-grade vectors, comprising the D64E IN mutant together with a conditional self-inactivating vector and a deleted PPT [45], as well as the development of a vector genome with the expression cassette in opposite orientation to the viral LTR, which reduces mobilisation of vectors [46].

## 4. Expression Levels of NILVs and Other Vector Designs

NILVs have been associated with lower expression levels in comparison to their integration-competent counterparts. It has been reported that these vector episomal DNAs are organised in nucleosomes and that modification of histones in these chromatin structures are typically associated with silenced chromatin, such as low levels of acetylation of H3 and H4, low level of dimethylation of H3-K4 and high levels of trimethylated H3-K9; importantly, the report also shows that HDAC inhibitors increased expression from episomal lentiviral vectors [47].

Expression levels may be important in certain applications of these vectors in clinical settings, where high expression of a transgene is required. Several approaches to increase expression could be explored, such as stronger promoter or enhancer elements, codon-optimisation techniques to improve translation of the desired transgenes, or the use of histone deacetylase inhibitors to activate transcription [48]. Other approaches include the removal of cis-acting elements from the U3 region of the LTR [49] or adding other cis-elements to enhance transcription, such as the IS2 element, a synthetic scaffold attachment region combined with the chicken haemoglobin HS4 insulator [47]. Yet another approach has been the use of viral proteins known to alter and optimise the host cell for increased transgene delivery and expression, such as Vpr [36] and Vpx [50,51], or the alteration of the cell conditions via drug treatment. Examples of the latter, although not specific for NILVs, include: target cell treatment with rapamycin, which enhances post-binding endocytic events via mammalian target of rapamycin (mTOR) inhibition [52]; treatment with caraphenol A, a cyclic resveratrol trimer that alters the levels of interferon-induced transmembrane (IFITM) proteins IFITM2 and IFITM3 and their association with late endosomes, thus augmenting lentiviral core escape from the endosomal compartment [53]; treatment with the pyrimidoindole derivative UM171 that acts via an unknown mechanism [54]. Although untested in the NILV format, the treatment of hematopoietic stem cells with Cyclosporine H augmented transduction of integrating lentiviral vectors by downmodulating IFITM3 [55], and, through a not yet identified mechanism, the use of PGE2 [56] or proteasome inhibitors [57,58] also increased transduction of lentiviral vectors. These may be other alternatives to increase the transduction efficiency of NILVs. 

Importantly, the mechanism by which vector episomal DNA results in lower expression of transgenes compared with integrated DNA is still unknown and warrants further investigation, potentially allowing the development of vectors that can circumvent this possible limiting step for the application of NILVs.

Other vector designs involved the use of NILVs to deliver genetic material that are then maintained episomally. This was accomplished by introducing the SV40 or EBV origins of replication in the NILV genome and the delivery of these vectors into cells expressing the SV40 large T antigen or the EBV nuclear antigen 1, respectively [59,60]. Alternatively, the use of a scaffold/matrix attachment region [61,62,63,64] or a transient induction of cell cycle arrest [65] also allows prolonged expression, even if the cells divide. Ultimately, safer gene addition therapies in dividing cells could be achieved by the use of NILVs to deliver the transgene that can then be the substrate of other integrating systems that display a safer integration profile compared to HIV-1 integrase—for example, the sleeping beauty transposase [66] or the Flp recombinase [67].

## 5. Old and Current Applications of NILVs

As discussed above, NILVs may have a safer profile than their integrating-competent counterparts, and when integration of the transgene is not necessary, for example, if the target cells are post-mitotic, the use of NILVs may be advantageous. Several studies have demonstrated sustained, long-term expression in many cell types and tissues, both in vitro and in vivo [37,40,68,69,70,71]. Additionally, NILVs have also demonstrated efficacy in pre-clinical models, such as retinal degeneration [37], Parkinson’s disease [72], Haemophilia B [73], or traumatic brain injury [74].

Other applications where non-integrating vectors may be important vectors of choice are those when transient expression is preferred. These include vaccination, cellular differentiation, delivery of cytotoxic cancer therapies, or as donor template delivery for homologous recombination and site-directed/specific integration systems.

Vaccination is an application where only transient expression is required and desirable. NILVs are therefore ideal candidates as vectors for this application and have been shown to elicit an efficient and sustained immune response both from intramuscular and sub-cutaneous injections, with prolonged CD8+ T cell responses and antibody production [75,76,77,78]. Several pre-clinical studies have shown the efficacy of NILVs in raising an immune response against HIV-1 [77,79,80,81,82], influenza virus [83,84,85], Zika virus [86], human papillomavirus [87], vaccinia virus [88], West Nile virus [89], human cytomegalovirus [90], Hepatitis B virus [75], and malaria [91]. NILVs have also been designed to specifically target professional antigen presenting cells, by using the Sindbis virus envelope glycoprotein that specifically targets DCs via the receptor DC-SIGN, which is expressed by these cells [92].

NILVs have also been utilised as vectors of choice for cancer immunotherapy [75,78,88,93,94,95,96]. These vectors have been successfully used to deliver immunogens to dendritic cells, professional antigen presenting cells that can directly prime CD8+ T cell responses. Of importance, one such vector has been now tested in the clinic. The LV305 vector has the mutation D64V in the viral integrase and is a dendritic cell-targeted vector that expresses the New York oesophageal squamous cell carcinoma-1 (NY-ESO-1) cancer testis antigen from a PPT-deleted vector genome. It was first reported as successfully treating a patient with metastatic and recurrent synovial sarcoma that was NY-ESO-1 therapy-refractive. This patient had been enrolled in a phase I dose-escalation clinical trial to evaluate the LV305 vectors. She received three doses of 5 × 10^8 vector genomes via intradermal injection showing no severe adverse events. Reportedly, the tumour in this patient shrunk 85% below baseline 24 months after LV305 injection. Importantly, the report shows sustained NY-ESO-1-specific CD4+ and CD8+ T cell responses that were sustained for up to two years after immunisation with the NILV [93]. In the first results of this clinical trial, it was reported that 39 patients had been enrolled, with tumour types including sarcoma, ovarian cancer, melanoma and lung cancer. The dose escalation ranged from three doses of 10^8 vector genomes to four doses of 10^10 vector genomes. Importantly, no dose-limiting toxicities were observed, and no patient reported a treatment-related severe adverse event. Secondary objectives of this trial included the assessment of immunogenicity. Despite the low number of enrolled patients, exploratory analysis showed that the disease control rate was 56%. Anti-NY-ESO-1 CD4+ and CD8+ T cell responses were observed in 57% of patients, and these responses were correlated with increased survival rate one-year post treatment [95].

Another study applied NILVs to treat cancer using a different approach, by turning NILVs into oncolytic viruses: NILVs expressing the diphtheria toxin A, an immunotoxin widely used in cancer treatment, under the control of the survivin promoter, a highly active promoter in tumour cells, were shown to inhibit tumour growth in immunodeficient nude mice [97]. 

One important area of scientific development is gene repair via endogenous homologous recombination. Here, NILVs have been used to deliver genetic material that can then be used as template for homologous recombination and thus gene editing of target cells [98]. However, the introduction of a double-strand break at the site of interest can enhance homologous recombination, and this can be achieved by the use of enzymes that specifically recognise target sequences, such as meganucleases and zinc finger nucleases (ZFNs). Lombardo and colleagues explored ZFNs to specifically integrate transgenes into the CCR5 and AAVS1 locus using NILVs. They demonstrated 7–12% efficiency of homologous recombination [99], while the iSCeI meganuclease was successfully utilised in gene repair in other reports [39,100]. Other groups showed pre-clinical efficiency of ZFNs in relevant disease models, such as adenosine deaminase deficiency [101], X-SCID [102,103], and β-thalassemia [104] or meganucleases for Duchenne muscular dystrophy [105].

The generation of induced pluripotent stem (iPS) cells is another area where transient expression is sufficient and desired and where NILVs have been tested. One study compared the generation of these cells by NILVs and their counterparts. It was found that, although NILVs were able to generate iPS cells, the efficiency was ~50-fold lower compared to their integrating counterparts, and that regardless of the vector system used, all generated clones had an integrated copy of the SV40 large T antigen gene (most likely arising from illegitimate integration in NILVs) [106]. However, sorting and identification of cells by transient expression of easily assessed transgenes by NILVs was shown to be effective [107,108].

## 6. Prospects for NILV Technology

The development of clustered regularly interspaced short palindromic repeats/CRISPR- associated protein 9 (CRISPR/Cas9) technologies that allow for the targeting of chromosomal DNA, introducing nicks or double-strand breaks at specific sequences and permitting precise editing of target DNA, opened a new avenue for the application of NILVs. Ideally, after the desired DNA editing event has occurred, Cas9 protein and guide RNA are not needed, and the genetic information should be deleted or lost from the cell to prevent off targets and undesirable side effects. Therefore, the use of a non-integrating vector is preferable, making NILVs highly attractive tools for this application: their genetic cargo is enough to accommodate the expression of the CRISPR/Cas9 protein as well as transcription of the guide RNA—necessary components for specific targeting and editing. The low immunogenicity of these vectors combined with the broad or specific cell targeting conferred by the choice of envelope pseudotyping is also advantageous. Indeed, it has already been demonstrated that these vectors are efficacious for such applications. In one study, HEK293T cells ectopically expressing green fluorescent protein (GFP) were transduced with NILVs expressing CRISPR/Cas9 and a guide RNA specifically targeting GFP to demonstrate the efficiency of this system. Additionally, this study measured on-target mutations and compared the results to integrating lentiviral vectors. The study found that on-target efficiency of NILVs was very similar to their integrating counterparts (80% compared to 84%, respectively). Importantly, off-target effects were also studied: while integrating lentiviral vectors were able to potentially introduce undesired mutations in 3.4 to 24% of 16 potential off-target genes, NILVs showed close to baseline mutations or only a slight increase of mutations relative to background in those genes [109]. NILVs have also been used to correct mutations in target cells, thus potentially ameliorating a disease. Surun and colleagues have shown that a non-integrating lentiviral vector expressing CRISPR/Cas9 and a guide RNA targeting the cytochrome b-245 heavy chain (whose lack of expression results in chronic granulomatosis disease) can be used to correct this defect in hematopoietic cells [110]. Izmiryan and colleagues corrected the gene responsible for recessive dystrophic epidermolysis bullosa, COL7A1, using an NILV design. Using an ex vivo approach, they were able to show successful correction of the gene in primary keratinocytes and fibroblasts. Of interest, this study also showed no non-specific cleavage of potential off-target hits and no expression of CRISPR/Cas9 one and two months after engrafting the corrected cells in mice [111]. Ortinski et al. targeted γ-amino-butyric acid A receptor subunit a2 by knocking out the expression of this protein and demonstrated the feasibility of this system in vivo in neurons [109]. Similarly, Hu and colleagues used NILVs to express CRISPR/Cas9 transiently in cells to introduce mutations in syngeneic cells that can then be transplanted in immunocompetent mice without the rejection of those cells. Using this technology, they reported the successful establishment of a von Hippel–Lindau gene-deleted metastatic renal cell carcinoma model in immunocompetent animals [112].

NILVs may also be the vectors of choice for the delivery of the DNA template necessary for homologous recombination. Importantly, it is plausible to speculate that there may be designs to allow for an all-in-one design to provide the DNA template sequence from the same vector that also expresses the Cas9 protein and guide RNA necessary to enhance this cellular process. This vector design would possibly increase the efficiency of these vectors to correct known mutations in the target DNA or to introduce known mutations to generate important disease model cells, in comparison to supplying the homology DNA template in a separate vector.

## 7. Conclusions

This review described the development of NILVs, achieved by mutating the viral integrase or the att sites in the viral DNA where this enzyme binds, and summarised the current and potential applications for these vectors. Originally, NILVs were developed to circumvent the potential insertional mutagenesis hazard posed by their integrating counterparts. It was shown that NILVs were able to sustain prolonged transgene expression in post-mitotic cells. Although lower expression levels are achieved by the episomal vector molecules compared with their integrating counterparts, several methods were applied to boost expression from those molecules. Nonetheless, further research should address this potential issue to elucidate this mechanism and guide other developments of these vectors.

The transient expression nature of NILVs in dividing cells, combined with their low immunogenicity and potential cell targeting through the use of pseudotyping and/or cell-specific promoters makes them ideal vector candidates to be used in applications that require temporary expression, such as vaccination, immunotherapies and the up-and-coming gene repair/editing techniques. With the revolutionary exploitation of CRISPR/Cas9 technology for easy genetic modification, including precise editing, it is conceivable that NILVs may be used as the delivery method of choice. NILVs’ intrinsic characteristics and their genetic cargo space that may be able to accommodate all required elements to achieve genetic editing (including the guide RNA, the Cas9 protein and, potentially, the DNA sequence to be used as template for genetic modification) are advantageous for the delivery of this system to target cells.

Overall, new avenues for applications of NILVs can be envisaged in the near future, so it is probable that we have not seen the last of these vectors yet.
